# Molecular Adaptations of BDNF/NT-4 Neurotrophic and Muscarinic Pathways in Ageing Neuromuscular Synapses

**DOI:** 10.3390/ijms25158018

**Published:** 2024-07-23

**Authors:** Marta Balanyà-Segura, Aleksandra Polishchuk, Laia Just-Borràs, Víctor Cilleros-Mañé, Carolina Silvera, Anna Ardévol, Marta Tomàs, Maria A. Lanuza, Erica Hurtado, Josep Tomàs

**Affiliations:** 1 Unitat d’Histologia i Neurobiologia (UHNeurob), Facultat de Medicina i Ciències de la Salut, Universitat Rovira i Virgili, Sant Llorenç 21, 43201 Reus, Spain; marta.balanya@urv.cat (M.B.-S.); aleksandra.polishchuk@urv.cat (A.P.); laia.just@urv.cat (L.J.-B.); victor.cilleros@urv.cat (V.C.-M.); carolina.silvera@urv.cat (C.S.); marta.tomas@urv.cat (M.T.); josepmaria.tomas@urv.cat (J.T.); 2 MoBioFood Research Group, Campus Sescelades, Universitat Rovira i Virgili, Marcel.lí Domingo 1, 43007 Tarragona, Spain; anna.ardevol@urv.cat

**Keywords:** motor endplate, acetylcholine release, muscarinic receptors, BDNF, TrkB, serine kinases, soluble N-ethylmaleimide-sensitive fusion protein attachment protein receptor (SNARE) complex

## Abstract

Age-related conditions, such as sarcopenia, cause physical disabilities for an increasing section of society. At the neuromuscular junction, the postsynaptic-derived neurotrophic factors brain-derived neurotrophic factor (BDNF) and neurotrophin 4 (NT-4) have neuroprotective functions and contribute to the correct regulation of the exocytotic machinery. Similarly, presynaptic muscarinic signalling plays a fundamental modulatory function in this synapse. However, whether or not these signalling pathways are compromised in ageing neuromuscular system has not yet been analysed. The present study analyses, through Western blotting, the differences in expression and activation of the main key proteins of the BDNF/NT-4 and muscarinic pathways related to neurotransmission in young versus ageing *Extensor digitorum longus* (EDL) rat muscles. The main results show an imbalance in several sections of these pathways: (i) a change in the stoichiometry of BDNF/NT-4, (ii) an imbalance of Tropomyosin-related kinase B receptor (TrkB)-FL/TrkB-T1 and neurotrophic receptor p 75 (p75^NTR^), (iii) no changes in the cytosol/membrane distribution of phosphorylated downstream protein kinase C (PKC)βI and PKCε, (iv) a reduction in the M2-subtype muscarinic receptor and P/Q-subtype voltage-gated calcium channel, (v) an imbalance of phosphorylated mammalian uncoordinated-18-1 (Munc18-1) (S313) and synaptosomal-associated protein 25 (SNAP-25) (S187), and (vi) normal levels of molecules related to the management of acetylcholine (Ach). Based on this descriptive analysis, we hypothesise that these pathways can be adjusted to ensure neurotransmission rather than undergoing negative alterations caused by ageing. However, further studies are needed to assess this hypothetical suggestion. Our results contribute to the understanding of some previously described neuromuscular functional age-related impairments. Strategies to promote these signalling pathways could improve the neuromuscular physiology and quality of life of older people.

## 1. Introduction

Motor neurons interact with skeletal muscle cells at the neuromuscular junction (NMJ), and the establishment of correct bidirectional communication is necessary to preserve the function of the synapse that must be maintained for proper muscle function in older individuals. Synapses are complex structures in which direct neurotransmission is constantly updated by several autocrine signals, for instance, presynaptic muscarinic control of acetylcholine (ACh) release, and by retrograde neurotrophic pathways, all of which provide synaptic plasticity to the system [1,2,3,4,5,6]. NMJ and muscles undergo important changes throughout life according to their plasticity capacities. The molecular plasticity associated with ageing has been poorly studied until now, although associated-signalling pathways are known to control the early dismantling of the skeletal muscle fibre and the NMJ. This is crucial in the onset of ageing, characterised by subsequent progressive motor weakness, skeletal muscle denervation and atrophy, and selective motor neuron death.

The postsynaptic-derived neurotrophic factors brain-derived neurotrophic factor (BDNF) and neurotrophin 4 (NT-4), acting through their receptors Tropomyosin-related kinase B receptor (TrkB) and neurotrophic receptor p 75 (p75^NTR^), have neuroprotective functions and enhance presynaptic downstream serine-threonine protein kinases (mitogen-activated protein kinase (MAPK), protein kinase C (PKC), and protein kinase A (PKA) isozymes) to maintain and regulate soluble N-ethylmaleimide-sensitive fusion protein attachment protein receptor (SNARE-SM) exocytotic proteins such as synaptosomal-associated protein 25 (SNAP25) and mammalian uncoordinated-18-1 (Munc18-1) [7,8], and therefore also regulate transmitter release. The full BDNF-NT4/TrkB-p75^NTR^/PKC-PKA/SNARE-SM pathway, which is essential to preserve the stability and functionality of the neuromuscular synapse [9,10,11,12]), can be positively modulated by activity-dependent adaptative changes. For instance, exercise strongly modifies this pathway in young muscles [13] and prevents its impairment in amyotrophic lateral sclerosis (ALS) muscles [14]. However, whether this neurotrophic signalling pathway is compromised or adapted in ageing neuromuscular systems has yet to be analysed.

Interacting with retrograde neurotrophic regulation, neurotransmission is also modulated by the muscarinic receptors present at the NMJ (M1 and M2) [15,16,17,18,19]. This autocrine pathway is relevant to balancing neuronal signalling, as M1 and M2 can enhance and decrease, respectively, the ACh release by regulating the phosphorylation of synaptic targets present in the SNARE-SM complex, which control vesicle docking and fusion to the membrane [20,21]. Furthermore, the voltage-gated calcium channels (VGCC) and the ACh reuptake complete the vesicle cycle, which is key to filling up ACh reservoirs inside vesicles and to collecting them and keeping them close to the presynaptic membrane. Unfortunately, whether or not ageing has some impact on muscarinic autoregulation and vesicle cycle steps has never been studied.

The present study analyses, using Western blotting, the effect of ageing on the expression (protein level) and activation (phosphorylation) of the main key proteins of the BDNF/NT-4, the muscarinic pathways, and the vesicle cycle molecules in the EDL rat muscle, which is a fast-twitch muscle susceptible to showing signs of ageing. At the myocellular level, many studies have reported a substantial decrease in muscle fibre size with age [22,23]. This reduction in muscle fibre size is fibre-type-specific, with 10–40% smaller type II fibres observed in the elderly compared to young adults. In contrast, type I muscle fibre size seems to be largely sustained with ageing [23,24,25]. EDL is what is called a fast-twitch muscle, since is composed mostly of fast-twitch fibres (≥70% Type IIB), which appear to be more prone to age-related alterations [26,27] due to their glycolytic metabolism.

Our results show that ageing has, firstly, a high impact on the neurotrophic pathway, resulting in a change in the stoichiometry of the BDNF and NT-4 neurotrophins, an unbalance of the receptors TrkB-FL/TrkB-T1, and a reduction in the active phosphorylated forms of the SNARE-SM complex proteins Munc18-1 (S313) and SNAP-25 (S187). Secondly, ageing induces downregulation changes in the M2 muscarinic pathway and in the P/Q-VGCC. Based on the descriptive analysis of the current study, we discuss the meaning of these changes and hypothesise that neurotrophic and muscarinic pathways can be adjusted for the promotion and saving of ACh release to ensure neurotransmission rather than undergoing negative alterations caused by ageing. Although there are alterations in some points of the relevant signalling pathways that regulate ACh release, it may be a fine and adaptable strategy to save neurotransmission activity. In a less positive interpretation, however, the evident downregulation of several neurotransmitter-release-related molecules may suggest the existence of a negative disturbance caused by ageing.

## 2. Results

### 2.1. Metrics of the Weights and Activity of the Animals and the EDL Muscle

**Figure 1:**
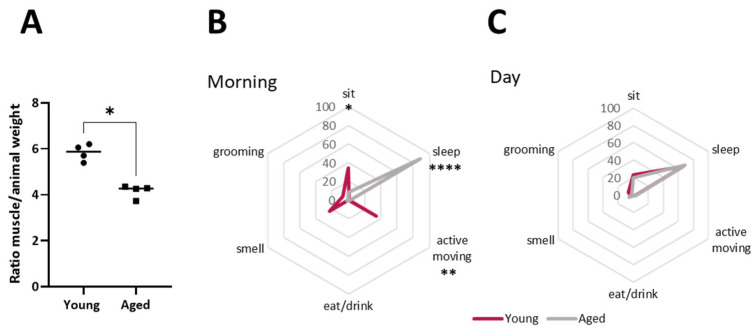
Metrics of weights and activity of rats and EDL muscle. (**A**), Representation of muscle weight normalised by animal body weight. (**B**,**C**), Spider graphs showing activity parameters during morning and afternoon time(s) of young and ageing rats. Data are represented as means ± SD. Statistical significance was determined using Mann–Whitney test (* *p* value < 0.05; ** *p* value < 0.01; **** *p* value < 0.0001).

Ageing rats (24 months) were compared with young adult rats (6 months) to analyse their weight and motility (Figure 1). There is no difference in the absolute EDL muscle weight between young and ageing animals. However, there is a significant difference in the EDL weight regarding the rat total weight (Figure 1A). Spider graphs in Figure 1B,C show that ageing rats spent more time sleeping during the morning and less time carrying out active movements, such as grooming and smelling.

### 2.2. Morphology of the Ageing NMJ

**Figure 2:**
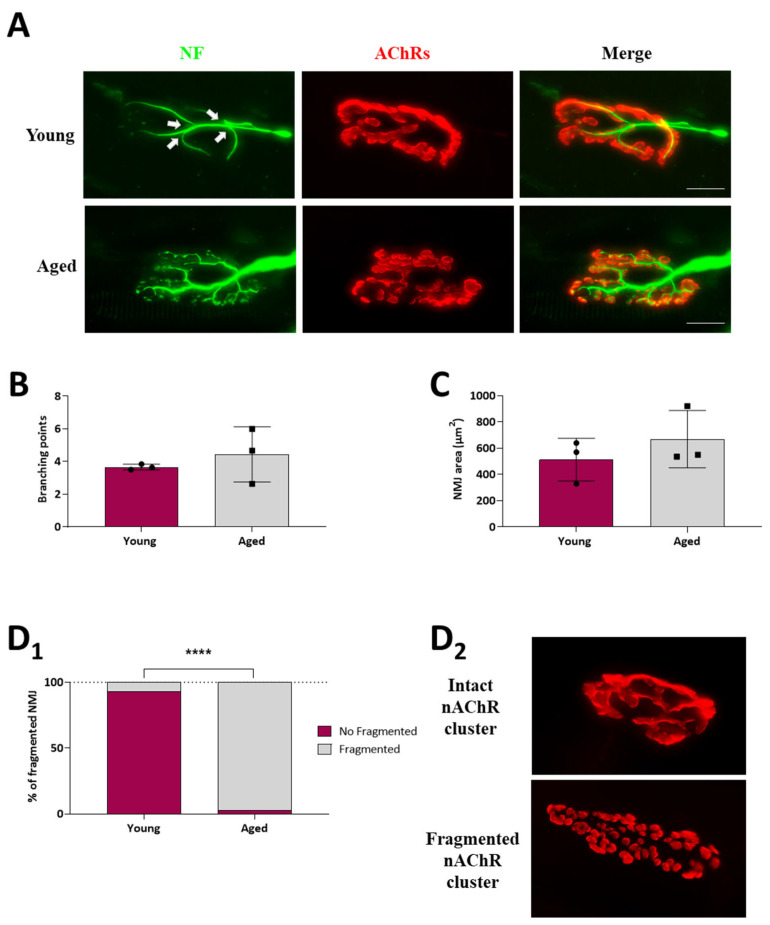
NMJ morphological analysis of young and ageing EDL muscles. (**A**), Immunofluorescence microscope images of representative NMJs from young and ageing EDL muscles, doubly labelled to identify the presynaptic nerve terminal (neurofilament, green), and the postsynaptic end plate (nAChRs; α-bungarotoxin, red). White arrows indicate the branching points. (**B**–**D_1_**), Histograms showing the quantification of morphological analysis. (**B**) Quantification of the number of branching points, (**C**) postsynaptic NMJ area, and (**D_1_**) NMJ fragmentation. Fragmented nAChR clusters are defined as five or more separated islands of nAChR clusters to form the endplate (previously described in [28]). Note that the percentages of fragmented NMJ significantly increase in ageing muscles. (**D_2_**) Representative images of intact and fragmented nAChR clusters showing how most young and ageing NMJs look, respectively. Scale bar: 25 µm. Data are represented as means ± SD. Statistical significance was determined using the Mann–Whitney test for graphs (**B**,**C**), and the graph (**D_1_**) was determined by 2-way ANOVA (**** *p* value < 0.0001). A minimum of 30 NMJs per rat from at least three separate rats of each group were quantified.

A quantitative morphological analysis of the immunohistochemical images was performed to examine the structural organisation of the ageing NMJ. We analysed some structural characteristics of the NMJs to determine the mean number of branching points in the nerve terminal, the mean postsynaptic area size, and whether it is fragmented or not. To label the pre- and postsynaptic components of the NMJ from EDL muscles via immunohistochemistry, we used an antibody, anti-neurofilament200, as a presynaptic marker to label motor nerve axons, and α-bungarotoxin, a selective and potent ligand for AChR, to visualise the postsynaptic membrane (Figure 2A). Even though some remodelling plastic changes normally occur in both the young and adult NMJ, we did not observe alterations in the nerve terminal branching points in ageing muscles (Figure 2B). However, the number of AChR-enriched membrane fragments in individual NMJs is higher in ageing muscles compared to young ones, as the percentage of fragmented NMJ in young muscles was 6.67 ± 3.3% compared to 97.23 ± 2.8% in ageing muscles (Figure 2D_1_,D_2_). Despite the increase in the number of fragments, there was no significant change in the total area occupied by these fragments (Figure 2C).

### 2.3. BDNF and NT-4 Neurotrophins

**Figure 3:**
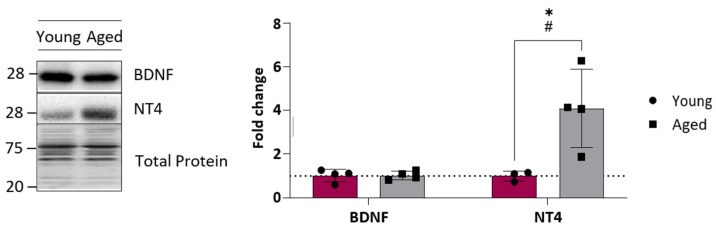
Neurotrophic factor levels of young and ageing EDL muscles. Western blot analysis of neurotrophins showed that BDNF protein levels do not change, but there is a significant increase in NT-4 protein levels in ageing EDL. Data are represented as means ± SD. Statistical significance was determined by Mann–Whitney test (* *p* value < 0.05). Statistical distribution significance is # *p* < 0.05.

The postsynaptic-derived neurotrophic factors BDNF and NT-4 trigger the neurotrophic pathways. Thus, the protein level of BDNF and NT-4 was analysed by Western blot (WB). Figure 3 shows that whereas BDNF did not change, a significant four-fold increase in the NT-4 level was observed in the ageing EDL muscle. Interestingly, an increased data dispersion was observed in ageing animals.

### 2.4. Neurotrophic Factor Receptors

**Figure 4:**
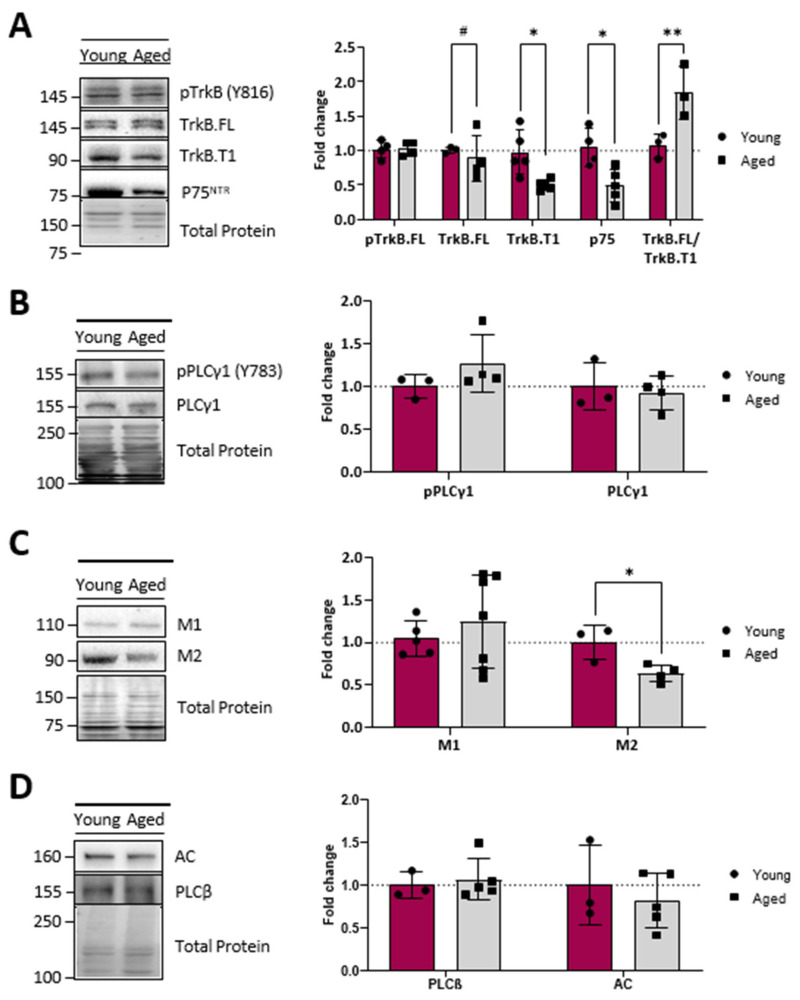
Neurotrophic factor receptor and muscarinic receptor levels of young and ageing EDL muscles. (**A**), Western blot analysis of neurotrophic receptors showing that TrkB-FL and its phosphorylated form, pTrkB-FL, do not change, but that there is a significant decrease in TrkB-T1 isoform and p75NTR receptor in ageing EDL. (**B**), Western blot analysis of PLCγ1 and p PLCγ1 shows no differences between young and ageing muscles. (**C**), Western blot analysis of muscarinic receptors, M1 and M2. (**D**), Western blot analysis of PLCβ and AC. Note that the protein level of M1 mAChR and PLCβ does not change with ageing but an important reduction in the M2 receptor occurs without affecting the membrane transducer adenylyl cyclase (AC). Data are represented as means ± SD. Statistical significance was determined using the Mann–Whitney test (* *p* value < 0.05; ** *p* value < 0.01). Statistical distribution significance is # *p* < 0.05.

Next, we analysed the protein level and phosphorylation of neurotrophins receptors. The WB analysis (Figure 4A) showed no age-related changes in either the level of TrkB-FL or in pTrkB-FL proteins. However, an important reduction (about 50%) in the TrkB-T1 isoform and p75^NTR^ receptor was observed. This favours the idea of an optimised TrkB-FL operation, resulting in less sequestration of the neurotrophins by TrkB-T1 and p75^NTR^ receptors. Interestingly, in concord with the stability of the protein level of the TrkB-FL and their active phosphorylated form, we found that its direct downstream transducer PLCγ (and its phosphorylated form) did not change in ageing EDL muscles (Figure 4B).

### 2.5. Muscarinic Receptors

In addition to the neurotrophic pathway, the NMJ is regulated by another signalling pathway, based on metabotropic receptors allowing communication between nerve terminals, postsynaptic muscle cells, and terminal glial Schwann cells [29]. Locally, neurotrophin receptors sense retrogradely derived signals that regulate presynaptic nerve terminals and transmitter release, while muscarinic receptors (mAChRs) enable the autocrine regulation of neurotransmission and plasticity through ACh release. Figure 4C,D show that the protein level of the ACh release enhancer, M1 mAChR, and its downstream transducer, PLCβ, does not change with age but, instead, there is a significant reduction in the M2 receptor (which downregulates ACh release) without affecting its membrane transducer adenylyl cyclase (AC). Thus, these data suggest that the M1/M2 ratio increase in ageing NMJ may facilitate ACh release, though the normal level of the first downstream enzymes favours a normalised transduction mechanism.

### 2.6. Protein kinases

**Figure 5:**
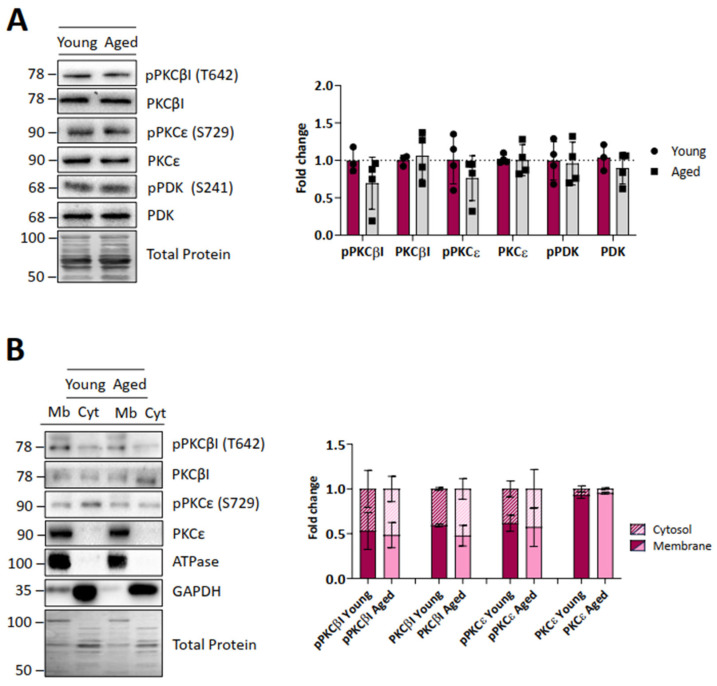
Protein kinase C and PDK levels of young and ageing EDL muscles. (**A**), Western blot analysis of different protein kinases and their phosphorylated forms. Quantification analysis shows that cPKCβI, nPKCε, and PDK1 protein levels (and their respective phosphorylated active forms) do not change in ageing muscles. (**B**), Distribution of the protein kinases between the membrane and cytosol fractions. PKCs are equally distributed in young and ageing muscles. Data are represented as means ± SD. Statistical significance was determined using the Mann–Whitney test.

The main downstream signalling pathway for the TrkB-FL receptor and mAChRs is mediated by some presynaptic isoforms [30]) of the serine-threonine kinase PKC. We observe here that the exclusive presynaptic isoforms cPKCβI and nPKCε and the priming kinase phosphoinositide-dependent kinase 1 (PDK1) (and their respective phosphorylated active forms) do not change in ageing animals (Figure 5A). These data are in accordance with the above-described stability of the upstream proteins TrkB-FL and PLCγ. Since pPDK1-primed PKCs are active when they translocate to the membrane, we also investigated the cytosol-membrane distribution of both cPKCβI and nPKCε (Figure 5B). The data indicate that in both young and ageing muscles, pcPKCβI, cPKCβI and pnPKCε are equally distributed between the membrane and cytosol, while nPKCε is mainly located in the membrane fraction. Therefore, these data suggest that there are no differences in the activation of PKCs between young and ageing muscles.

**Figure 6:**
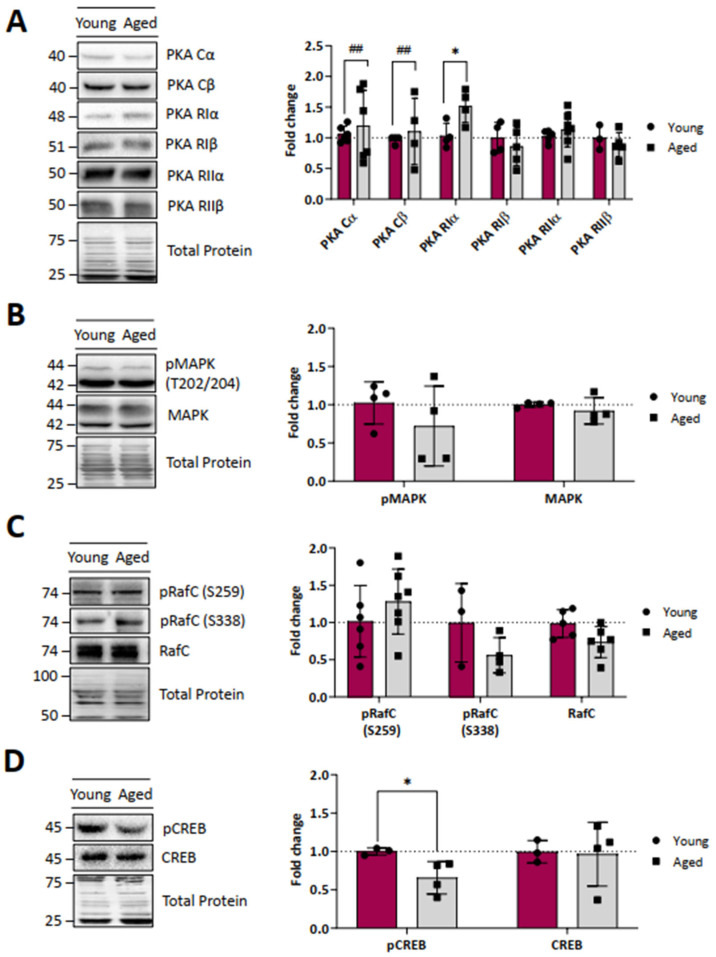
Protein kinase A subunits’ levels of young and ageing EDL muscles. (**A**–**C**), Western blot analysis of different PKA subunits (**A**), MAPK (**B**), and RAfC proteins (**C**), showing that the protein levels of almost all proteins do not change in ageing muscles. Note that there is an increase in the protein levels of PKA RIα. (**D**), Western blot analysis of CREB and its phosphorylated form shows a reduction in pCREB in ageing muscles. Data are represented as means ± SD. Statistical significance was determined using the Mann–Whitney test (* *p* value < 0.05). Statistical distribution significance is ## *p* < 0.01.

Next, we analysed the protein levels of different catalytic and regulatory subunits of PKA, which are also regulated by TrkB and muscarinic receptors at the NMJ [21,31]. We found no change in the mean value of the protein level of the PKA catalytic alpha and beta and the regulatory subunits RI beta and RII alpha and beta (Figure 6A). However, we observed an increase in the protein levels of PKA Riα, suggesting a significant compensatory regulation of PKA activity. In addition, an increase in the variability in ageing muscles can be observed, similar to other molecules previously described.

The transcription factor cyclic AMP response element-binding protein (CREB) is a PKA target that is an important regulator of BDNF because pCREB regulates the BDNF gene itself. Here, we observed a significant reduction in the pCREB protein level in ageing animals. These data would be related to the observed change in the BDNF/NT-4 ratio in older animals (Figure 6D).

Finally, we evaluated another set of kinases regulated by neurotrophic and muscarinic pathways such as RafC and MAPK. Young and ageing muscles showed no difference in any of these proteins (Figure 6B,C).

### 2.7. Kinase Targets in the SNARE-SM ACh Release Complex

**Figure 7:**
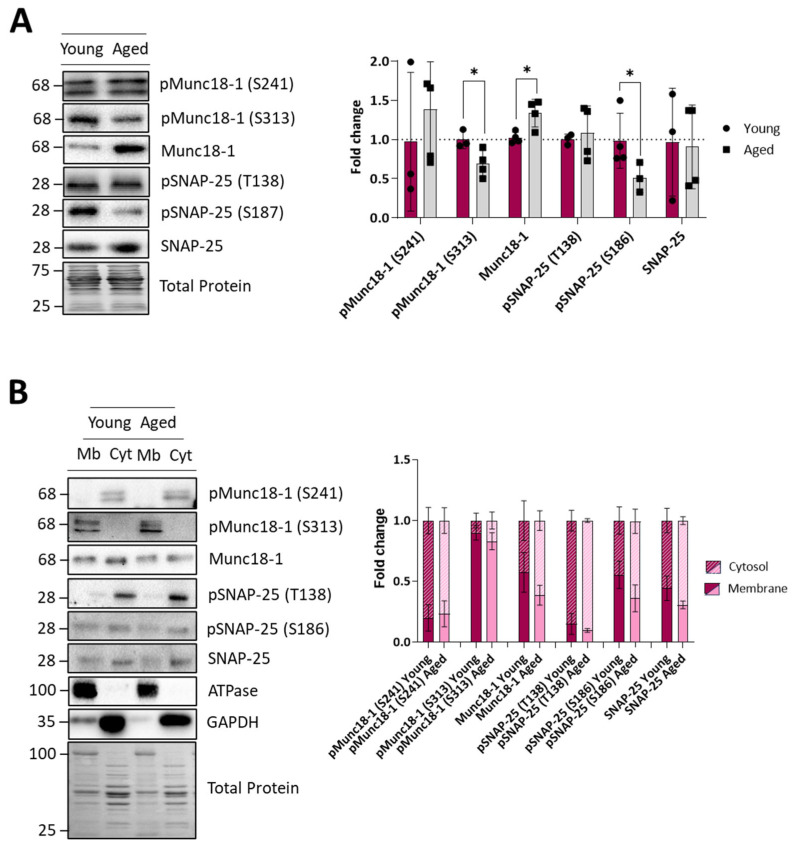
Protein levels of key molecules related to the SNARE-SM ACh release complex of young and ageing EDL muscles. (**A**), Western blot analysis of different protein kinases targets and their phosphorylated forms. Quantification analysis shows a decrease in phosphorylated Munc18-1 and an increase in Munc18-1 protein levels in ageing muscles. The SNAP-25 protein level does not change as the PKA-dependent pSNAP-25 (T138) does. However, the cPKCβI- and nPKCε-dependent pSNAP-25 (S187) are greatly reduced in ageing. (**B**), The distribution of the protein kinases targets between the membrane and cytosol fractions shows no differences between young and ageing muscles. Data are represented as means ± SD. Statistical significance was determined using the Mann–Whitney test (* *p* value < 0.05).

To assess whether the ACh release machinery of ageing rats is affected, we investigated the protein and phosphorylation levels of two relevant proteins of the presynaptic SNARE-SM complex, the regulatory Munc18-1 and the structural SNAP-25 (Figure 7). We found that ageing muscles express lower PKC-induced pMunc18-1 (S313) and a normal level of MAPK-induced pMunc18-1 (S241), accompanied by an increase in total Munc18-1 protein levels. The SNAP-25 protein level does not change as the PKA-dependent pSNAP-25 (T138). However, the cPKCβI- and nPKCε-dependent pSNAP-25 (S187) is greatly reduced in ageing (Figure 7A). The decrease in the phosphorylated Munc18-1 (S313) and SNAP-25 (S187) could suggest a defective mechanism in their phosphorylation by PKC. Interestingly, however, we could not discern any difference in the membrane and cytosol distributions of all these proteins between young and ageing muscles (Figure 7B).

### 2.8. Acetylcholine Cycle Proteins and Calcium Channels

**Figure 8:**
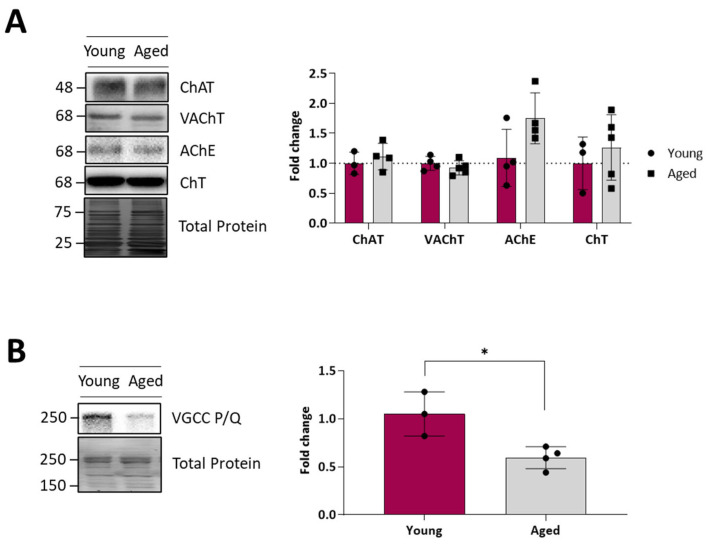
Synaptic vesicles and calcium channel levels of young and ageing EDL muscles. (**A**), Western blot analysis of different key presynaptic molecules related to synaptic vesicles. Note that no change was found in any protein analysed. (**B**), Western blot analysis of the P/Q-type VGCC, showing a significant decrease in the protein levels between young and ageing muscles. Data are represented as means ± SD. Statistical significance was determined using the Mann–Whitney test (* *p* value < 0.05).

Finally, we analysed the protein levels of calcium channels and key presynaptic molecules related to the ACh availability in synaptic vesicles to evaluate possible alterations to the synaptic vesicle cycle. Figure 8A shows no change for the choline transporter of the presynaptic membrane (ChAT), the vesicular membrane acetylcholine transporter (VChAT), acetylcholinesterase (AChE), and choline acetyltransferase (ChT). This suggests the correct management of ACh in the synaptic vesicle cycle in ageing motor nerve terminals.

Finally, in ageing animals, we observed a strong diminution of the P/Q type voltage-gated calcium channel (VGCC), suggesting that there could be an alteration in the coupling of neuronal excitation to the secretion of neurotransmission through this channel (Figure 8B).

**Figure 9:**
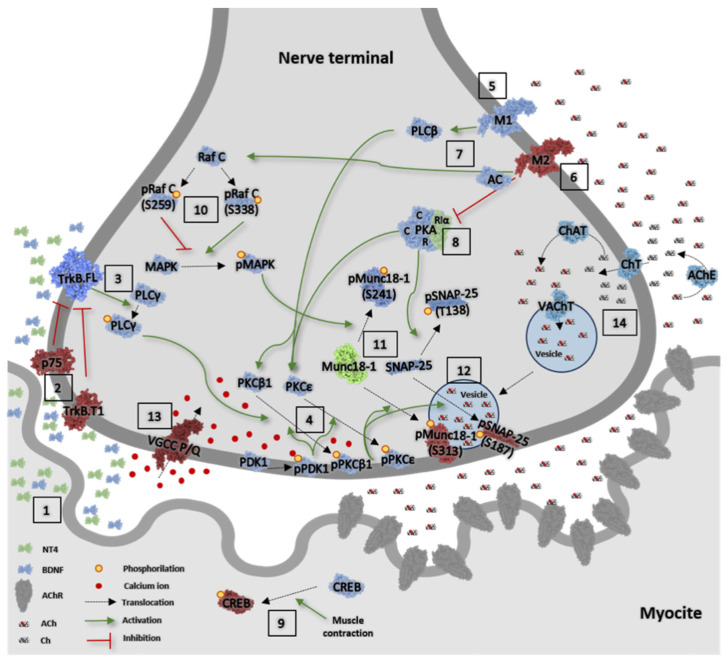
Overview of the main molecular elements of the neurotransmission signalling altered in ageing EDL muscles. Image information: Protein levels of those molecules represented in (1) green are increased, (2) in red are decreased, and (3) in blue are maintained in ageing muscles compared with young muscles. Related to the neurotrophic pathway, #**1**, there is a change in the stoichiometry of the BDNF and NT-4 neurotrophins, because of the increased levels of NT-4. **#2**, TrkB.T1 protein levels are decreased, increasing the TrkB.FL/T1 ratio, and p75^NTR^ is also decreased. **#3**, TrkB.FL direct downstream transducer PLCγ (and its phosphorylated form) do not change in ageing EDL muscles. **#4**, The protein level and the cytosol/membrane distribution of the presynaptic phosphorylated downstream PDK and PKCβI and PKCε isoforms is maintained in ageing muscles. **#5**, The protein levels of M1-subtype muscarinic receptor are maintained, and **#6** the protein level of the M2-subtype muscarinic receptor is reduced. **#7**, The levels of the M1 mAChR downstream transducer PLCβ and M2 mAChR downstream transducer adenylyl cyclase are maintained. The only change observed relative to its downstream PKA kinase, #**8**, is the increased level of the PKA subunit RIα. As stated, the upregulation of this regulatory subunit may be linked to the reduction in pCREB in the postsynaptic site, **#9**. **#10**, cRaf and MAPK, kinases regulated by neurotrophic and muscarinic pathways, are unaffected by ageing. Both PKC and PKA regulate the SNARE-SM ACh release complex. **#11**. There is an imbalance in the SNARE-SM complex proteins Munc18-1 and SNAP-25 (and their phosphorylated forms). Although pMunc18-1 phosphorylated in S241 does not change, there is a decrease in pMunc18-1 (S313) and an increase in Munc18-1 protein levels in ageing muscles. **#12**, The SNAP-25 protein level does not change, or the PKA-dependent pSNAP-25 (T138). However, the cPKCβI- and nPKCε-dependent pSNAP-25 (S187) is greatly reduced in ageing. Despite these modifications, there is no difference in the membrane and cytosol distributions of these proteins between young and ageing muscles. Finally, **#13**, although there is a strong diminution of the P/Q type voltage-gated calcium-channel (VGCC) in ageing, **#14**, all proteins related to ACh and recycling synaptic vesicles (acetylcholinesterase, choline transporter in the plasmalemma, cholin acetyl transferase, and vesicular acetylcholine transporter) are unmodified in ageing muscles.

Figure 9 represents the main molecular elements of the neurotransmission signalling analysed here, showing the observed ageing changes.

## 3. Discussion

Here, we investigate the existence of age-related molecular changes in the BDNF/NT-4 neurotrophic and muscarinic signalling, which represent the most noticeable modulatory and neuroprotective pathways of the neuromuscular system. Our question is: are these signalling pathways compromised in the ageing neuromuscular system and its synapses? If they are, these changes could represent a deleterious ageing disturbance or a functional adjustment in response to usage modifications to ensure neurotransmission. We analysed the morphology of the NMJ, and we did not observe alterations in the nerve terminal branching points in ageing muscles. However, there was an increased fragmentation of AChR clusters, which would indicate that there is an active remodelling process. Despite the increase in the number of fragments, there was no significant change in the total area occupied by these fragments. A prominent morphological change in the rodent NMJ during ageing is the fragmentation of the post-synaptic AChR clusters [28,32], although the sequence of events that results in the fragmentation of NMJs has yet to be fully revealed. Since fragmentation coincides with muscle weakness and is reversed by anti-sarcopenic interventions such as exercise and caloric restriction [28], fragmentation has often been taken as a sign of NMJ dysfunction. However, other studies suggest that the progressive fragmentation of individual NMJs, such as that which occurs naturally with age, does not correlate with a decline in the efficacy of neuromuscular transmission at those NMJs, and has even been described as the enhancement of neuromuscular transmission with age [33,34,35,36]. Thus, considering our results, the NMJ remodelling signs observed in the ageing NMJ may be interpreted as an adaptative or reparative mechanism to maintain muscle function. We also observed some activity-related behavioural changes in the ageing animals, such as a moderate lengthening of the morning sleep period. It has been demonstrated that physical activity has different effects on the NMJ and skeletal muscles, leading to structural, molecular, and functional adjustments [37,38,39]. However, we think that this small decrease in activity would not influence the molecular phenotype of neurotrophic signalling.

With age, the progressive loss of skeletal muscle mass that occurs is considered a key factor in the decline in physical performance in older people. The sarcopenia index (SI) is evaluated as a muscle weight/body weight (GMW/BW) ratio. When the SI value of the rats from the ageing group is significantly lower than that of the young group, sarcopenia could be considered [40]. Here, we observed a decrease in the EDL muscle weight related to the total corporal weight, suggesting evidence of sarcopenia in ageing animals. However, the EDL weight was not decreased in these females, ageing rats, and further experiments are needed to evaluate muscle loss. The increase in total corporal weight in ageing rats has been associated with the onset of obesity and related diabetogenic situations [41], but it seems that there is not a compromise of the NMJ functionality, as many of the proteins studied are unchanged in these rats.

As both BDNF/TrkB and muscarinic signalling are essential to modulate NMJ maintenance and promote neurotransmission, here, we investigated the entire TrkB and muscarinic pathways at the synapses of EDL muscles at their three molecular levels: (i) the neurotrophic factors BDNF and NT-4 and their receptors, TrkB full length (TrkB.FL) and truncated (TrkB.T) and p75^NTR^; (ii) the coupled serine-threonine kinases, PKC isoforms, and their priming kinase PDK1, the different subunits of the PKA, and the MAPK; (iii) PKC, PKA, and MAPK targets related to ACh release (Munc18-1 and SNAP-25 and CREB); and acetylcholine cycle proteins and calcium channels. Changes are represented in the intuitive graphic in Figure 9 in the nerve terminal of the NMJ, where, despite some factors being expressed in the extra-synaptic region (BDNF, NT-4, TrkB, p75, PKA, MAPK), most of the factors studied are exclusively localised in the presynaptic area. This spatial characteristic of the pathway is extremely useful for understanding the localisation and age-related changes in muscle tissue and attributing downstream final changes located in the nerve terminal of the NMJ. It can be noted that the cellular environment of the muscle that changes during ageing can also be related to non-muscle and non-neuronal cells such as Schwann cells, endothelial cells, immune cells, FAPs, etc. This would be an issue for further research.

### 3.1. Neurotrophins

The BDNF/NT-4/TrkB pathway is the main retrograde signalling pathway involved in NMJ stability and is essential to regulate neurotransmission [42,43,44,45]. BDNF and NT-4 are strongly expressed in the skeletal muscle, in particular at the NMJ, in response to muscle activity [46,47]. We found in the ageing EDL muscles a clear increase in the NT-4 (four-fold) without change in the BDNF protein level, indicating a good production of BDNF and an overproduction of NT-4. We hypothesise that a slow motor neuron recruitment might be happening in ageing EDL as a consequence of NMJ ageing adaptation, which is in concord with other studies that evidence that NT-4 seems to be a molecule characteristic for the proper functioning of slow fibre types [13,14,48], and type I muscle fibres seem to be largely sustained with ageing [23,24,25]. Further experiments staining for MyHC types to show age-related changes in myofiber type would help in our understanding of this question.

### 3.2. Neurotrophin Receptors

BDNF and NT-4 selectively bind and activate the specific receptor TrkB [2,43,49,50]. Alternative splicing generates one TrkB full-length isoform (TrkB-FL), with an intracellular kinase domain, and two truncated isoforms (TrkB-T1 and TrkB-T2) that do not have it [51,52]. TrkB-FL, when it is not inhibited through TrkB-T1 heterodimerisation [53,54,55,56,57,58,59], activates its downstream signalling via trans- and autophosphorylations in the intracellular domain of the receptor and the subsequent activation of cytoplasmic signalling pathways including phospholipase Cγ (PLCγ) and Ras-MAPK. In addition, it has been shown that muscle contraction downregulates TrkB-T1 and increases TrkB-FL [42,60]. Our results show that TrkB-FL and pTrkB-FL, along with their downstream transducer PLCγ, protein levels, and phosphorylated forms, did not change, while TrkB-T1 and p75^NTR^ receptors diminished in the ageing animals. This balance of neurotrophic receptors, along with the increase in NT-4 and preservation of BDNF levels, provides a good molecular configuration to guarantee downstream signalling in the ageing EDL muscle.

Concerning the p75^NTR^ receptor, we observed an important reduction (about 50%) in the protein level in the ageing EDL muscle. p75^NTR^, a member of the tumour necrosis factor receptor family [61,62], binds to all neurotrophins with similar affinity [63]. Its cytoplasmic domain transmits very complex downstream signalling to determine whether neurons survive or not during development [64]. Also, it has been shown that the ability of NT-4 to activate TrkB are negatively regulated by high levels of p75^NTR^ [65,66,67], thus modulating the responsiveness to neurotrophins, similar to the effect of TrkB-T1. The low protein level in ageing EDL muscles of both TrkB-T1 and p75^NTR^ receptor seems optimised to favour TrkB-FL signalling, which balances this system to plasticity and neuronal survival instead of apoptosis or degeneration.

### 3.3. Muscarinic Receptors

Muscarinic receptors have a functional link to TrkB receptors performing a cooperative mechanism controlling ACh release in the presynaptic compartment [68]. M1 and M2 muscarinic receptor subtypes induce opposed outcomes on ACh release at the NMJ, with M1 increasing it, whereas M2 decreases the end-plate potential [69,70]. Here, we observe an important reduction in M2 receptor protein in ageing NMJs without a change in the M1 receptors. This could indicate an age-induced downregulation of the mechanism that prevents ACh release, as M2 signalling decreases the PKA phosphorylation of SNAP-25 T138 at the NMJ [21]. However, in the ageing NMJ, there is no change in the AC level or the phosphorylation of PKA target SNAP-25 T138, as would be expected as M2 receptors are linked to Gi proteins, which inhibit AC and block PKA activity by downregulating cAMP production [71,72]. Downregulated M2 and maintained downstream proteins indicate M2 as a key molecule to be affected early in age to favour strategies to maintain the neurotransmitter release. One of these strategies could favour M1-induced signalling, which is what the results show, as we observe an increase in the M1/M2 ratio in the ageing NMJ. M1 signalling enhances ACh release by using Gq/PLCβ to promote the phosphorylation of PDK1 and the priming and maturation of the presynaptic PKC isoforms cPKCβI and nPKCε to phosphorylate at least the SNARE regulator Munc18-1 (Ser313) and the SNARE core protein SNAP-25 (Ser187) [20]. Here, we show that almost all this signalling pathway is maintained in ageing EDL muscles.

### 3.4. Downstream Protein Kinases

Both receptors, TrkB and M1/M2, trigger signalling pathways that confluence on kinases that modulate neurotransmitter release. It is well established that pTrkB-FL, through PLCγ, generates IP3 and DAG that results in calcium release from intracellular stores that activate several PKC isoforms [73] through PDK1 [74]. Of particular interest are cPKCβI and nPKCε (and their primer kinase PDK), which are exclusive to the NMJ presynaptic nerve terminal and essential for ACh release [42,75]. Here, we found that cPKCβI, nPKCε, the primer kinase PDK1, and their respective phosphorylated forms do not change in ageing animals, indicating a regular activation of these kinases in ageing. Furthermore, their distribution between cytosol and membrane is also unchanged, enhancing the idea of a correct functionality, as primed PKCs are translocated to the membrane for the further activation and phosphorylation of targets [74].

PKA also regulates ACh release by phosphorylating synaptic targets thanks to the action of its six different subunits. Regulatory subunits (RIα, RIβ, RIIα and RIIβ) anchor the catalytic ones (Cα and Cβ), assembling an inactive tetramer. Once catalytic subunits have been released from the holoenzyme, as a response to cAMP binding to the regulatory subunits they become active and able to phosphorylate downstream targets [71,72,76]. In addition to cAMP activation, PKA is also activated by the subcellular targeting of the subunits [77,78] and by changes in its concentration [79,80]. Here, we found that the RIα subunit is increased in ageing animals, and it could anchor catalytical subunits to prevent PKA’s further phosphorylation. RI is more efficient than RII in inducing CREB response, regardless of the C subunit [81,82,83], and here we found a decrease in CREB phosphorylation, which might be related to the rise in the RIα subunit. In addition, RIα has demonstrated its capacity for the significant compensatory regulation of PKA activity in tissues where the other regulatory subunits are expressed, including the brain, brown and white adipose tissue, skeletal muscle, and sperm [84]. Therefore, our results reveal two mechanisms by which PKA would be downregulated in ageing MMJ: the decrease in M2 and the rise in RIα. The decrease in M2 seems insufficient to cancel the presynaptic activity of catalytic subunits, as there is a stable level of phosphorylation of at least one presynaptic PKA target (pSNAP (T138)), surely because there is a balance between M1 and M2, where M1 increases its activity when M2 decreases it [21]. This is in concord with the unchanged values of AC in ageing EDL NMJs. However, it should be taken into account that the cellular environment of the muscle changes during ageing and we cannot discard the fact that the observed variations could originate from non-muscle and non-neuronal cells such as Schwann cells, endothelial cells, immune cells, or FAPs.

cRaf and MAPK are another set of kinases partially regulated by neurotrophic and muscarinic pathways [85,86,87]. G protein-coupled receptors, such as muscarinic receptors, activate the downstream Ras protein, which in turn activates cRaf, and this latter activates MAPK, leading to a final substrate phosphorylation (in this case, S241 pMunc18-1). The phosphorylation of cRaf at residue S338 triggers its activation and the subsequent MAPK phosphorylation cascade [88]. In contrast, cRaf phosphorylation in S259 prevents the activation of the MAPK/ERK pathway [89]. We assume that this pathway is not affected by ageing as there are no differences in any of the proteins related to the MAPK pathway.

In summary, all downstream kinase pathways seem to work optimally, although there are some changes in their upstream receptors and downstream targets. Due to this event, we cannot relate the changes seen in the receptors to the changes seen in the exocytotic machinery, although our laboratory has previously shown that synaptic targets are regulated by neurotrophin and muscarinic receptors in an activity-dependent manner. However, we cannot discard the possibility that changes downregulating vesicle exocytosis (discussed below) might impact the upregulation of the retrograde signalling of neurotrophic and muscarinic pathways.

### 3.5. Presynaptic Molecular Machinery of Transmitter Release

Some of the PKC, PKA, and MAPK targets are proteins of the exocytotic machinery at the NMJ presynaptic nerve terminal, such as Munc18-1 and Synaptosomal nerve-associated protein 25 (SNAP-25) [7,8].

#### 3.5.1. SNAP-25

SNAP-25, synaptobrevin, and syntaxin, are the three SNARE proteins of the core fusion vesicle complex which is involved in vesicle docking, priming, and the triggering of fast exocytosis [90,91]. SNAP-25 is phosphorylated by PKC in serine 187 [35], and by PKA in threonine 138 [92]. Whereas T138 phosphorylation controls the size of the releasable vesicle pools, the S187 phosphorylation of SNAP-25 enhances the recruitment after the releasable vesicle pools have been emptied [93]. At the NMJ, these phosphorylations are activity-dependent and regulated by PKA and PKC, respectively [8,31,74]. Furthermore, the correct balance between both SNAP-25 phosphorylations is needed, which is regulated by muscarinic [20,21] and neurotrophic receptors () to ensure an accurate neurotransmission process [8,31,78,92,93]. In the ageing EDL muscle, PKC-dependent pSNAP-25 (S187) is greatly reduced while the pSNAP-25 T138 protein level does not change. We hypothesise that some reduction in synaptic vesicle recruitment to the active zones can be expected after the releasable vesicle pools have been emptied (pSNAP-25 S187 reduction levels), although the size of the releasable vesicle pools would be normal (normal pSNAP-25 T138 levels).

#### 3.5.2. Munc18-1

PKC phosphorylates Munc18-1 in S313 [94,95,96,97], while the MAPK pathway phosphorylates it in S241 [98]. The ratio of PKC/MAPK phosphorylating activity on the regulatory protein of vesicular release Munc18-1 can regulate exocytosis, as increased PKC phosphorylation in S313 favours exocytosis, whereas increased MAPK phosphorylation in S241 reduces ACh release. In ageing EDL NMJs, Munc18-1 is less phosphorylated in S313 residue, while S241 phosphorylation is not changed, which would mean that the ratio is balanced to a reduction in vesicle release. TrkB and M1 control pMunc18-1 (S313) through cPKCβI and nPKCε [7,21]. However, in ageing NMJs, only the final target of the signalling pathway, pMunc18-1 (S313), is decreased, indicating an additional control on this phosphorylation affected by age. Total Munc18-1 is more abundant in ageing compared to in young individuals, which could be related to accumulation or upregulation to compensate for lower phosphorylation.

Moreover, we observed that M2 mAChRs positively regulate the MAPK pathway and the phosphorylation of S241, contributing to the effect of M2 in reducing ACh release. Even though M2 is downregulated, it does not seem to impact the MAPK pathway leading to S241 Munc18-1 phosphorylation in ageing, as neither change their abundance levels. The observed reduction in M2 protein may involve the prevalence of PKC phosphorylation.

### 3.6. Presynaptic Acetylcholine Cycle Proteins and Calcium Channels

To evaluate possible alterations to the synaptic vesicle cycle, we analysed the protein level of key presynaptic molecules related to synaptic vesicles and calcium channels. Acetylcholine (ACh) is synthesised in nerve terminals from choline and acetyl coenzyme A by the cytoplasmic enzyme choline acetyltransferase (ChAT) [99]. We also evaluated the vesicular acetylcholine transporter (VAChT) that is responsible for loading ACh into secretory vesicles, and acetylcholinesterase (AChE), which is a cholinergic enzyme primarily found in postsynaptic neuromuscular junctions, especially in muscles and nerves. It immediately breaks down or hydrolyses acetylcholine (ACh), a naturally occurring neurotransmitter, into acetic acid and choline [100]. Finally, the presynaptic choline transporter (ChT) is essential for providing choline as a substrate for the synthesis of ACh. The protein level of these molecules showed no change in the ageing EDL muscle. Firstly, these data indicate that there was not a relevant loss or degeneration of motor nerve terminals. Secondly, there was apparently good management of the ACh in the ageing motor nerve terminals without important alterations to synaptic vesicle recycling.

However, we found a significant diminution of the P/Q-type VGCC protein level in the ageing EDL muscle. A reduction in the protein level per synapse without a change in the morphologically identified puncta density has also been found [101]. The presence and specific functions of P/Q-type VGCC are identified, along with others, like L- and N-types, in the motor nerve terminals at the NMJ during development [102] and regeneration [103].

Because P/Q-type VGCC allows activity-dependent calcium entry that promotes ACh release in the adult NMJ, defective neurotransmission can be expected in these ageing muscles. In relation to synaptic transmission parameters, ageing-related changes are described. In several muscles, including EDL, the miniature end-plate potential (mEPP) frequency (presynaptic in origin) diminishes with no change in its amplitude [33]. There is also an increased amplitude of the first evoked end-plate potential (EPP) of a train (and the quantal content, presynaptic in origin), followed by decreased amplitude thereafter [33,104]. These electrophysiological changes suggest a relevant age-related adaptation of the ACh release probability and in the releasable synaptic vesicle number and turnover. Regarding a plausible direct involvement of the P/Q channel reduction and calcium inflow in age-related release changes, our results, found in this article, fit quite well, as S186 pSNAP-25 and S313 pMunc18-1, two key proteins in vesicle docking and fusion, are downregulated.

## 4. Materials and Methods

### 4.1. Animal Model

Wistar rats obtained from Envigo (Barcelona, Spain) were used for this experiment. We evaluated the *Extensor digitorium longus* (EDL) muscle from 6 month-old and 24 month-old rats. They were housed individually at a room temperature of 23 °C with a standard 12 h light–dark cycle, ventilation, and ad libitum access to a standard chow diet and tap water. This procedure was approved by the Experimental Animal Ethics Committee of the Generalitat de Catalunya, Spain (Department of Territory and Sustainability, General Directorate for Environmental and Natural Policy, project authorisation code: 10183). For each type of experimental condition, at least three animals (*n* ≥ 3) were used as biological replicates.

### 4.2. Video Processing for Rat Behaviour Analysis

The video monitoring of rats’ behaviour was performed during one hour for 7 days. Two sessions of recording were conducted: morning video monitoring (between 8 a.m. and 10 a.m.) and afternoon video monitoring (between 12 p.m. and 14 p.m.). We used a video camera, Sony Handycam HD, for recording rats’ activity. It allowed the monitoring of several cages of rats at the same time. To estimate the activity of each group of rats, the zoom was used for each frame cage. The analysis of the animal behaviour was not carried out by blinding the investigator, since the phenotypes of the young and ageing rats are very different. To differentiate all types of activity, 6 patterns of behaviour were allocated: sitting, sleeping, actively moving, eating/drinking, smelling, and grooming.

### 4.3. Sample Processing

#### 4.3.1. Whole Cell Lysate

Animals were weighed and euthanised when they reached their specific age (either 6 or 24 months), and then EDL muscles were extracted, weighed, and deep-frozen using liquid nitrogen. To perform the Western blot technique, muscles were homogenised using a VWR VDI 12 homogeniser in an ice-cold lysis buffer (in mM: NaCl 150, Tris-HCl (pH 7.4) 50, EDTA 1, NaF 50, PMSF 1, sodium orthovanadate 1; NP-40 1%, Triton X-100 0.1%, and protease inhibitor cocktail 1%) (Sigma-Aldrich, Saint Louis, MO, USA). Protein lysates were obtained by collecting supernatants after removing insoluble materials through centrifugation at 4 °C, and aliquots were stored at −80 °C. Protein concentrations were determined by DC protein assay (Bio-Rad, Hercules, CA, USA).

#### 4.3.2. Membrane/Cytosol Fractionated Lysates

Samples were immediately homogenised without freezing to avoid membrane damage before purification. The lysis buffer was prepared without detergents (in mM: NaCl 150, Tris-HCl (pH 7.4) 50, EDTA 1, NaF 50, PMSF 1, Na3VO4 1; and protease inhibitor cocktail 1%). First, homogenised samples were centrifugated at 1000× *g* for 15 min at 4 °C to remove insoluble materials. The pellet was discarded, and the resulting supernatant was further centrifuged at 130,000× *g* for 1 h. The new supernatant corresponded to the cytosolic fraction, while the pellet corresponded to the membrane fraction. The membrane fraction was then resuspended in lysis buffer (in mM: NaCl 150, Tris-HCl (pH 7.4) 50, EDTA 1, NaF 50, PMSF 1, Na_3_VO_4_ 1; NP-40 1%, Triton X-100 0.1%, and protease inhibitor cocktail 1%). The purity of the subcellular fractionation was determined with the cytosol-specific GAPDH and the membrane-specific Na^+^/K^+^-ATPase.

### 4.4. Western Blot

Protein lysates were obtained by collecting supernatants after removing insoluble materials through centrifugation at 4 °C, and aliquots were stored at −80 °C. Protein concentrations were determined with a DC protein assay (Bio-Rad, Hercules, CA, USA). Protein samples of 30 µg were separated by electrophoresis using an 8% or 12% SDS-polyacrylamide gel and electro-transferred to a polyvinylidene difluoride (PVDF) or a nitrocellulose membrane. Membranes were blocked for an hour, and then they were incubated in primary antibody overnight. Finally, membranes were incubated with a corresponding secondary horseradish peroxidase-conjugated antibody for one hour. Since each primary antibody has its own specifications regarding membrane, blocking solution, concentration, and secondary antibody, they are summed up in Table 1.

Membranes were revealed with a Bio-Rad ECL kit on the ChemiDoc XRS+ machine (Bio-Rad, Hercules, CA, USA). The integrated optical density of the bands was normalised with respect to (1) the background values and (2) the total protein transferred on membranes, measured by total protein analysis (Sypro Ruby protein blot stain, Bio-Rad) [105]. Relative variations between samples were calculated from the same membrane image. Data were taken from densitometry measurements made in at least three separate Western blots.

### 4.5. Immunohistochemistry

EDL muscles were dissected and embedded in 15% sucrose until they sank and then embedded in 30% sucrose until they sank as well. After that, they were fixed in 4% paraformaldehyde for 40 min at room temperature. Lastly, they were embedded in OCT compound (Ref. 00411243, VWR), frozen in isopentane precooled with liquid nitrogen, and stored at −80 °C. Serial sections 40 µm thick were cut in a cryostat, collected on SuperFrost^®^Plus microscope slides (Ref. 631-0108, VWR), and stored at −80 °C.

Sections were permeabilised with 1% Triton X-100 in phosphate-buffered saline (PBS), and nonspecific binding was blocked with 4% BSA for 1 h. Then, sections were incubated overnight with neurofilament primary antibody at 4 °C (1/500; Ref. N5389, Merck). The next day, they were rinsed and incubated for 4 h at room temperature with A488 secondary antibody (1/1000; Ref. T1175, Molecular Probes) and α-bungarotoxin-TRICT (1/1000; Ref: A21202, Life Technologies). Finally, sections were rinsed and mounted with Mowiol. The secondary antibody specificity was tested by incubation in the absence of a primary antibody.

Slides were visualised using a Leica DMI 6000 B microscope. The maximal intensity projection of z-stack images was reconstructed using the ImageJ software 1.48v version (Wayne Rasband; National Institutes of Health, Bethesda, MD, USA), and several parameters were analysed to determine the morphology of the NMJs.

The branching point number of the nerve terminals was counted, as it indicates the arborisation pattern complexity. Fragmented nAChRs are defined as five or more separated islands of nAChR clusters to form the endplate (previously described in Valdez et al., 2010). To quantify the area of the NMJs facing forward, the region occupied by nAChRs, labelled by BTX, was measured. A minimum of 30 NMJs per rat from at least three separate rats of each group was quantified.

### 4.6. Statistical Analysis

All values were represented as mean ± standard deviation (SD) within each group, and each dot represents the value of one animal to visualise their distribution.

The normality of the distributions was tested with a Shapiro–Wilk test. The statistical significance of the differences between means of both experimental groups was evaluated using the Mann–Whitney test. Differences between SD were evaluated using the F test (GraphPad Prism software 9, San Diego, CA, USA). The criterion for statistical mean significance was * *p* < 0.05, ** *p* < 0.01, and *** *p* < 0.001, and distribution significance was # *p* < 0.05, ## *p* < 0.01, and ### *p* < 0.001.

## 5. Conclusions

Molecular elements of neurotransmission signalling analysed here are represented in Figure 9. We investigated whether neuroprotective BDNF/NT-4 signalling and muscarinic signalling are compromised in ageing neuromuscular synapses. We found relevant changes in the protein level and the phosphorylation of certain key proteins associated with the presynaptic membrane, and changes in neurotrophic and muscarinic pathways that may be adaptative effects to save future neurotransmission activity, rather than detrimental.

The main results show an imbalance in different sections of the signalling pathway, from neurotrophic and muscarinic signalling to the acetylcholine neurotransmitter release cycle. 

Related to the neurotrophic pathway, there was a change in the stoichiometry of the BDNF and NT-4 neurotrophins, because of increased levels of NT-4. We think that this change may be linked to the reduction in pCREB, probably associated with the increase in RIα PKA regulatory subunits in the postsynaptic site. These neurotrophins bind to neurotrophic receptors in the nerve terminal that are also unbalanced. TrkB.T1 protein levels are decreased, increasing the TrkB.FL/T1 ratio, and p75^NTR^ also decreases. The downregulation of T1 and p75^NTR^ favours the functionality of the maintained FL isoform and its downstream signalling, including the transducer PLCγ (and its phosphorylated form), the cytosol/membrane distribution of the presynaptic phosphorylated downstream PDK, and PKCβI and PKCε isoforms.

Other proteins that allow the autocrine regulation of neurotransmission are muscarinic receptors (mAChRs). The increase in the M1/M2 ratio, because of the reduction in the protein level of M2-subtype muscarinic receptor in ageing NMJ, may facilitate ACh release because of the well-known reciprocal function of these receptors.

Therefore, the results show that the “negative” partners of the neurotrophic (T1 and p75) and muscarinic (M2 type) receptors are downregulated in ageing NMJs, suggesting a molecular adaptation of the machinery to ensure enough neurotransmission in the ageing nerve terminals.

Moreover, ageing NMJs show decreased levels of several phosphoproteins involved in the exocytosis of the synaptic vesicles, including PKC-dependent pMunc18-1 (S313) and PKC-dependent pSNAP-25 (S187), but not MAPK-dependent pMunc18-1 (S241) or PKA-dependent pSNAP-25 (T138). These results would mean that the ratio of phosphorylations is balanced with a reduction in vesicle release. In addition, P/Q-type VGCC is also strongly decreased. Together, these molecular changes are in accordance with the ACh release probability changes that have been described in ageing muscles.

In summary, in the ageing NMJs, there is an adaptation of neurotrophins (BDNF/NT-4 ratio decrease), neurotrophin receptors (TrkB.FL/T1 ratio), and autocrine muscarinic receptors (M1/M2 ratio increase) that can be coupled to (1) the downstream PKC isoforms (PKCβI, PKCε and PDK) which have normal protein levels, phosphorylation, and distribution; (2) the PKA catalytic and regulatory subunits (with a reduction in RIα), and (3) MAPK. Similarly, the molecular pathway allowing choline recapture, ACh synthesis, and vesicle refilling appears to be normal in ageing muscles. However, a relevant downregulation in the serine phosphorylation of the active zone SNARE-SM proteins Munc18-1 (S313) and SNAP-25 (S187) coincides with a decrease in the P/Q calcium channel protein level. Thus, some mismatch between the well-adapted signalling receptors and kinases on one side and the vesicular recruitment and calcium-dependent exocytosis on the other side may be related to the ACh release probability changes that have been described in ageing muscles. However, functional and physiological experiments are needed to fully understand these signalling pathways in ageing.

## Figures and Tables

**Table 1:** Antibody summary.

Target	kDa	Origin Antibody	Reference	Dilution	Blocking Solution	Membrane	Family	
VGCC P/Q-Type CaV2.1	250	Rabbit polyclonal	ACC-001	1/1000	milk	PVDF	Calcium channels	
BDNF	14/32	Rabbit polyclonal	28205-1-AP	1/1000	milk	PVDF	Neurotrophics	
NT4	14	Rabbit polyclonal		1/500	milk	PVDF		
p75	75	Rabbit polyclonal	07-476	1/1000	milk	PVDF		
pTrkB (Y816)	145	Rabbit polyclonal	Novus NBF1-03499	1/1000	BSA	PVDF	TrkB receptors	
TrkB	95/145	Rabbit polyclonal	4603S 80E3	1/1000	BSA	PVDF		
PLCβ	155	Mouse monoclonal	sc-5291	1/1000	BSA	PVDF	PLCs	
pPLCy1(Y^783^)	155	Rabbit polyclonal	2821S CST	1/800	BSA	Nitrocellulose		
PLCy1	155	Mouse monoclonal	sc-7290	1/1000	milk	PVDF		
M1 mAChR	100	Rabbit polyclonal	AMR-001	1/1000	milk	PVDF	Muscarinics	
M2 mAChR	90	Rabbit polyclonal	AMR-002	1/1000	milk	PVDF		
p-Raf-C (Ser^259^)	74	Rabbit polyclonal	9421 CST	1/1000	BSA	PVDF	MAPK pathway	
p-Raf-C (Ser^338^)	74	Rabbit monoclonal	9427 CST	1/1000	BSA	Nitrocellulose		
Raf-C	65–75	Rabbit monoclonal	9422 CST	1/1000	BSA	PVDF		
pMAPK/ERK (Thr202/204)	42	Rabbit polyclonal	9101 CST	1/1000	BSA	PVDF		
MAPK/ERK	42	Rabbit polyclonal	9102 CST	1/1000	BSA	PVDF		
pPDK1 (Ser241)	58–68	Rabbit polyclonal	CST (3061)	1/1000	BSA	PVDF	PKCs	
PDK1	58–68	Mouse monoclonal	sc-17765	1/1000	BSA	Nitrocellulose		
pPKCß1 (Thr642)	76	Rabbit polyclonal	ab5782	1/1000	BSA	PVDF		
PKCß1	76	Mouse monoclonal	sc-8049	1/1000	milk	PVDF/Nitro		
pPKCƐ (Ser729)	90	Rabbit polyclonal	sc-12355	1/1000	BSA	PVDF		
PKCƐ	90	Rabbit polyclonal	sc-214	1/1000	milk	PVDF		
PKA Cα	40	Mouse monoclonal	sc-28315	1/1000	milk	PVDF	PKAs	
PKA Cβ	40	Rabbit polyclonal	sc-904	1/1000	milk	PVDF		
PKA RIα	48	Mouse monoclonal	sc-136231	1/1000	milk	PVDF		
PKA RIβ	51	Rabbit polyclonal	sc-907	1/800	milk	Nitrocellulose		
PKA RIIα	50	Rabbit polyclonal	sc-909	1/1000	milk	PVDF		
PKA RIIβ	53	Rabbit polyclonal	ABS-14	1/800	milk	Nitrocellulose		
Adenylate Cyclase	160	Rabbit polyclonal	PA5-35382	1/1000	BSA	Nitrocellulose	AC	
pMunc18-1 (Ser^241^)	68	Rabbit polyclonal	Ab183484	1/1000-1/700	BSA	Nitrocellulose/PVDF	Target of MAPK pathway	Munc18-1 (SM)
pMunc18-1 (Ser313)	68	Rabbit polyclonal	ab138687	1/1000	p-Block	PVDF	Target of PKA	
Munc18-1	68	Rabbit polyclonal	CST (D406V)	1/1000	milk	PVDF		
pSNAP-25 (Ser187)	28	Rabbit polyclonal	ab169871	1/1000	BSA	PVDF	Target of PKC	SNAP-25 (SNARE)
pSNAP-25 (Thr^138^)	28	Rabbit polyclonal	orb163730	1/1000	BSA	PVDF	Target of PKA	
SNAP-25	28	Rabbit polyclonal	CST (5309)	1/1000	BSA	PVDF, Nitrocellulose		
pCREB (Ser^133^)	43	Rabbit polyclonal	CST (9191S)	1/1000	BSA	PVDF	Target of PKA, p90RSK, MSK, CaMKIV, and MAPKAPK-2	CREB (bZIP transcription factor that activates target genes)
CREB	43	Rabbit polyclonal	CST (9192)	1/1000	milk	PVDF		
GAPDH	37	Mouse monoclonal	sc-32233	1/2000	milk	PVDF	Marker of cyt	Markers of mb and cyt
ATPase	112	Mouse monoclonal	DSHB (a6f)	1/1000	milk	PVDF	Marker of mb	
CHAT	48	Rabbit polyclonal	207471AP	1/1200	milk	Nitrocellulose	Synaptic vesicle cycle	
AChE	68	Goat poly	ab31276	1/1000	BSA	Nitrocellulose		
VAChT	68	Rabbit polyclonal	SAB4200559	1/300	BSA	Nitrocellulose		
Secondary antibody		Donkey polyclonal	711-035-152	1/10,000	-	-		
Secondary antibody		Rabbit polyclonal	A9044	1/10,000	-	-		

Primary antibodies used and their commercial reference, respective membrane, blocking solution, concentration used, the family they belong to, and secondary antibodies. Abbreviations: AC: Adenyl cyclase; AChE: Acetylcholinesterase; ATPase: Adenosine triphosphatase; BDNF: Brain-derived neurotrophic factor; BSA: Bovine serum albumin; ChAT: Choline acetyltransferase; CREB: CRE- binding protein; cyt: Cytosol; ERK: Extracellular signal-regulated kinase; GAPDH: Glyceraldehyde-3- Phosphate Dehydrogenase; M1 mAChR: Muscarinic acetylcholine receptor M1; M2 mAChR: Muscarinic acetylcholine receptor M2; MAPK: Mitogen-activated protein kinase; mb: membrane; Munc18-1: Mammalian homologue of uncoordinated-18; NT4: Neurotrophine 4; PDK1: 3-phosphoinositide-dependent kinase 1; PKA: Protein kinase A; PKC: Protein kinase C; PLC: Phospholipase C; PVDF: Polyvinylidene difluoride; SNAP-25: Synaptosomal-associated protein of 25 kDa; TrkB: Tyrosine kinase B; VAChT: Vesicular acetylcholine transferase; VGCC: Voltage-gated calcium channel.

## Data Availability

Data is contained within the article.

## Abbreviations

AC: adenylyl cyclase; ACh, acetylcholine; ALS, amyotrophic lateral sclerosis; AR, adenosine autoreceptors, BDNF, brain-derived neurotrophic factor; CREB, cyclic AMP response element-binding protein; EDL, *Extensor digitorum longus*; EPP, evoked endplate potentials; nAChRs, nicotinic acetylcholine receptors; mAChR, muscarinic acetylcholine receptor; M_1_, M_1_-type muscarinic acetylcholine receptor; M_2_, M_2_-type muscarinic acetylcholine receptor; M_4_, M_4_-type muscarinic acetylcholine receptor; NMJ, neuromuscular junction; NT-4, neurotrophin 4; PKA, protein kinase A; PKC, protein kinase C; TrkB, tropomyosin-related kinase B receptor; VGCC, voltage-gated calcium channels; WB, Western blot.
